# Arresting the Activity of Bacterial β-Barrel Pore-Forming Toxins by Cysteine Insertion Mutagenesis in the Homologous Region

**DOI:** 10.3390/ijms27083590

**Published:** 2026-04-17

**Authors:** Alexander V. Siunov, Bogdan S. Melnik, Alexey S. Nagel, Zhanna I. Andreeva-Kovalevskaya, Natalia V. Rudenko, Anna P. Karatovskaya, Olesya S. Vetrova, Anna V. Zamyatina, Fedor A. Brovko, Alexander S. Solonin

**Affiliations:** 1 FSBIS FRC Pushchino Scientific Centre of Biological Research, G.K. Skryabin Institute of Biochemistry and Physiology of Microorganisms, Russian Academy of Sciences, 5 Prospekt Nauki, 142290 Pushchino, Russia; av_siunov@rambler.ru (A.V.S.); hemolysin6@gmail.com (Z.I.A.-K.); 2 Institute of Protein Research, Russian Academy of Sciences, 4 Institutskaya Street, 142290 Pushchino, Russia; bmelnik@phys.protres.ru; 3 Pushchino Branch, Shemyakin–Ovchinnikov Institute of Bioorganic Chemistry, Russian Academy of Sciences, 6 Prospekt Nauki, 142290 Pushchino, Russia; nrudkova@mail.ru (N.V.R.); annakaratovskaya@mail.ru (A.P.K.); olesja.wetrowa1999@gmail.com (O.S.V.); anna.zamjatina@yandex.ru (A.V.Z.); brovko@bibch.ru (F.A.B.)

**Keywords:** bacterial β-barrel pore-forming toxins, Hla, HlyII, CytK-2, disulfide bond formation, hemolytic activity, monoclonal antibodies, mouse model

## Abstract

Bacterial β-barrel pore-forming toxins, including *Staphylococcus aureus* α-toxin (Hla) and *Bacillus cereus* toxins hemolysin II (HlyII) and cytolytic toxin K2 (CytK-2), are secreted by bacterial cells as water-soluble monomers. These monomers assemble within lipid bilayers to form cylindrical pores, leading to lysis of target eukaryotic cells. We created mutant forms of these toxins that, based on the results of X-ray structural analysis of Hla and the prediction of the 3D structure of HlyII and CytK2, can form intramolecular disulfide bonds in monomers. The substitutions were made in the region responsible for toxin insertion into the target membrane. The mutant forms reversibly altered their hemolytic activity depending on the presence of reducing reagents and were non-toxic when injected into experimental animals. The immune response to injection of the mutant forms of Hla and CytK-2 toxins resulted in higher antibody titers against the wild-type toxins and a higher level of immunological memory than with injection of the HlyII mutant. The mutant form of CytK-2 demonstrates the properties of a prototype vaccine, as immunization with this protein protects animals against the effects of the wild-type toxin.

## 1. Introduction

Bacterial toxins belonging to the β-barrel pore-forming protein family form pores in artificial and natural bilayer membranes [1] in several steps. All β-barrel pore-forming toxins (β-PFTs) are secreted from bacterial cells as water-soluble monomers and assemble in the lipid bilayer of target cell membranes, forming oligomeric membrane pores [2]. In many cases, the assembly and incorporation of oligomeric membrane proteins proceed via complex pathways that may involve multiple accessory proteins. These accessory proteins play an important role in pore formation, complicating the elucidation of the precise molecular mechanisms involved [3,4].

The most studied bacterial β-PFT is the α-toxin of *Staphylococcus aureus* (Hla) [5]. More than 10,000 papers have been published since it was first studied in 1929 [6,7]. The three-dimensional structures of both the monomeric and oligomeric forms of this toxin have been determined. Structural studies of the water-soluble Hla monomer have revealed conformational changes upon the conversion of the monomer to the oligomeric form [8,9]. Although the atomic structure of the final assembly step has been determined [10] and key amino acid residues essential for the assembly have been identified, the molecular mechanisms underlying oligomerization and membrane integration of toxins still remain unclear. This is largely due to the difficulty of obtaining structural information on the short-lived intermediate steps of the assembly process [11,12].

In 1993, a new hemolytic activity of *Bacillus cereus* was described [13] and attributed to hemolysin II (HlyII). The presence of a signal peptide ensures the secretion of this protein from bacteria in the form of water-soluble monomers. In the presence of membranes of susceptible cells, the monomeric forms in the membranes of these cells assemble mainly into heptamers, forming transmembrane pores. Water-soluble monomers undergo significant conformational changes when converted to membrane-bound oligomers. HlyII belongs to the β-PFT family and contains a 94-amino-acid C-terminal extension that has no homology with proteins of this family [14]. The remaining 284-amino-acid portion (excluding the signal peptide and C-terminal extension) shares a 28–31% identity with Hla.

Another hemolytic protein of this family in *B. cereus* is cytolytic toxin K2 (CytK-2). The amino acid sequence of CytK-2 is 37% identical to that of HlyII and 30% identical to that of Hla [15]. These toxins possess a region of high homology in the domain involved in the interaction with the biological membrane. Monoclonal antibodies to the domain formed by the C-terminal extension of HlyII have been previously obtained [16]. The use of these antibodies makes it possible to partially identify conformational changes during the transition from the monomeric to the oligomeric form [16,17].

HlyII, like Hla, is capable of forming mainly heptameric structures [18], although pores can also assemble as hexamers or octamers [19,20]. Assembly intermediates are short-lived in nature, and methods to delay the transition from monomeric to oligomeric forms of the toxin at specific stages of pore-forming have been proposed. It has been previously demonstrated that introducing cysteine residues into Hla can arrest pore maturation at intermediate stages [21,22,23,24,25].

Pore-forming toxins play a key role in infectious processes caused by bacterial pathogens. Previously, fragments of these toxins lacking hemolytic activity have been used to produce neutralizing antibodies capable of suppressing the hemolytic activity of pore-forming toxins [16,26,27,28,29,30]. Mutations that suppress cytolytic activity, such as the H35A substitution in Hla [31], and deletion mutants, in which 39 amino acids are replaced by four amino acids (PSGS) [32], have also been employed. In the latter case, the mutant forms of Hla are capable of forming inactive heptameric forms.

In an infected organism, β-PFTs trigger a variety of cellular reactions that are significant for the infection process and the host immune response [33]. Damage to cell membranes caused by β-PFTs often leads to the release of cytokines that activate immune cells. For example, β-PFTs have been shown to induce the production of IL-6, one of whose functions is to stimulate the proliferation and differentiation of B cells [34].

Full-length β-PFTs are recognized by the immune system as protein antigens. The specific immunogenicity of β-PFTs practically depends on a variety of factors, including the toxin structure and the individual host response [35]. The mechanisms of cellular defense, or the immune response, remain largely unclear. Investigating various defense strategies and understanding their activation mechanisms could facilitate the development of therapeutics against the destructive effects of β-PFTs.

In this study, we described full-length mutant forms of three bacterial toxins, Hla, HlyII and CytK-2, with introduced double cysteine residues and demonstrate that rearrangements in the homologous region [28] affect the lytic activity of the toxins. We investigated their toxic effects, the level of immune response to injection of the mutant forms of the toxins, and the neutralizing properties of immune sera to analyze the potential use of the resulting mutants as vaccine prototypes.

## 2. Results

### 2.1. Design of the Double-Cysteine Mutants

**Figure 1:**
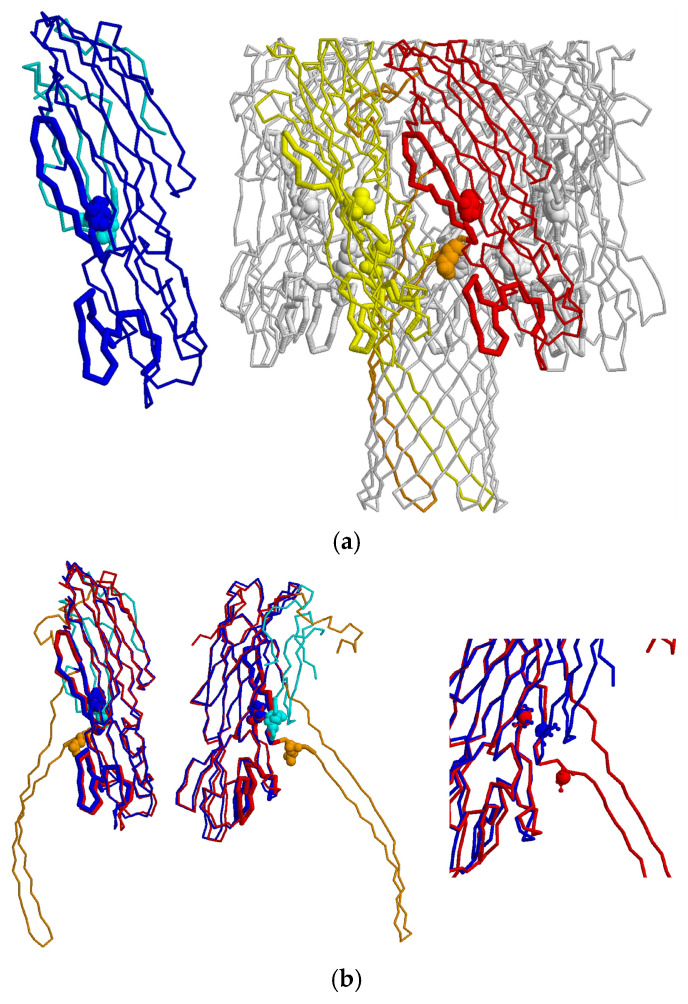
Structure of Hla. (**a**) Left panel: Structure of the monomeric soluble form of Hla (PDB: 4YHD); right panel: structure of the oligomeric form of Hla (PDB: 3ANZ); (**b**) superposition of one subunit of the monomeric and oligomeric forms of Hla. The monomeric form is shown in blue; the oligomeric form, in gray. The subunits of the oligomeric form are highlighted in yellow and red. Regions of the protein that change conformation during the transition from the monomeric to the oligomeric form are shown in light blue and orange. Conserved regions in the alignment of HlyII and CytK-2 from *B. cereus* are shown as thickened main chains. Glutamine and valine amino acid residues, selected for substitution with cysteines, are shown in 3D form. The right panel of (**b**) shows an enlarged fragment of the superimposed subunits. The C_β_ atoms of the selected amino acid residues are indicated by larger spheres. It can be seen that in the structure of the monomeric soluble form the C_β_ atoms of the selected amino acids (blue spheres) are oriented toward each other and located very close together, whereas in the structure of the oligomeric form, the C_β_ atoms (red spheres) are far apart (14 Å).

Prediction of the relative positions of individual amino acid residues in the monomeric and oligomeric forms of Hla allowed us to design and generate double-cysteine mutants within the homologous region [28]. To design mutations that could affect conformational rearrangements of the protein, we used models of the 3D structures of Hla in monomeric (PDB: 4YHD) [36] and oligomeric forms (PDB: 3ANZ) [25]. The 3D structures of the protein subunit in the soluble monomeric and oligomeric forms were compared. A superposition of these structures is shown in Figure 1. It is evident that upon conversion of the soluble monomer into the toxic oligomer, the subunit structure changes at the protein’s N-terminus and in the loop that forms a transmembrane channel. The rest of the subunit structure either remains unchanged or changes only slightly.

**Figure 2:**
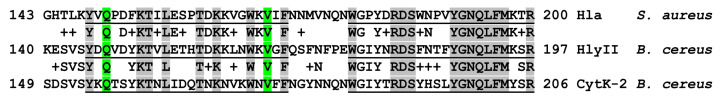
Pairwise alignment of amino acid sequences of hemolysin HlyII with CytK-2 from *B. cereus* and Hla from *S. aureus*, generated using the BLAST tool (https://blast.ncbi.nlm.nih.gov/Blast.cgi, accessed on 15 February 2026) [37]. Regions with high homology are underlined. Residues that are identical among all three toxins are shaded in gray. The symbol “+” indicates conservative substitution. The glutamine and valine residues replaced by cysteines in the mutant constructs are highlighted in green.

To design a disulfide bridge between cysteines that would lock the monomeric form and prevent oligomerization, we applied two criteria. First, the two residues to be replaced should be located close to each other in the monomer of the protein but sufficiently far apart in the oligomer. Second, these positions should be located within a region conserved among the β-PFTs Hla [36], HlyII [14], and CytK-2 [28]. Figure 1b (right panel) shows that the C_β_ atoms of the selected amino acid residues are located very close together in the soluble form of the subunit (blue structure in Figure 1) and sufficiently far apart in the structure of the subunit forming the oligomeric protein. Figure 2 shows the alignment of the amino acid sequences of Hla (Uniprot: Q2G1X0), CytK-2 (Uniprot: Q81GS6), and HlyII (Uniprot: Q81AN8) and shows that two regions (underlined in Figure 2) possess high homology.

However, only one region containing the amino acid pair Q150 and V167 (numbering from the PDB structure: 4YHD) in the Hla protein meets both criteria: in the monomeric form, the C_β_ atoms of Q150 and V167 are located at a distance of less than 6 Å, whereas in the oligomeric form these atoms are at a distance of about 14 Å. This is clearly visible in the right panel of Figure 1b. Thus, we hypothesized that introducing the Q150C and V167C substitutions into Hla would lock the monomeric soluble form of the protein, thereby preventing formation of the toxic oligomer in the absence of SH reagents. In addition, AlphaFold modeling of the HlyII and CytK-2 structures [38,39] showed that the selected amino acids were located in the triangle region [40], and the stem regions were formed by the most conserved hydrophobic residues and small side chains. These regions play an important role in the conformational rearrangements of β-barrel pore-forming proteins during pore formation and are involved in interactions between monomers during the conversion of the water-soluble form of the toxin to the oligomeric membrane-bound form [41]. Considering the high degree of homology in this region among Hla, HlyII and CytK-2, we expect that HlyII Q147C/V166C and CytK-2 Q156C/V175C, designated as HlyIICM and CytK-2CM respectively, will exhibit similar properties to those of Hla Q150C/V167C, designated as HlaCM, and their hemolytic activity will depend on the presence of SH reagents in the reaction mixture.

In their natural state, all toxins considered in this study lack cysteine residues, allowing cysteine to be used to replace amino acid residues in polypeptides within the region of high homology between the toxins. Based on the significant similarity in the primary sequences and comparative analysis of the secondary structures of Hla, HlyII, and CytK-2, we generated double-cysteine mutants HlyIICM, HlaCM, and CytK-2CM. The mutant toxins were stable upon overexpression. A minor fraction was found in the water-soluble fraction and exhibited reduced hemolytic activity, while the majority of the toxins were in the insoluble fraction of cell debris.

We created three double-cysteine mutants capable of forming disulfide bonds. The HlyII mutant contained six histidine residues at the N-terminus and a TEV protease cleavage site. The CytK-2 mutant had six histidine residues at the C-terminus and a thrombin cleavage site. The Hla mutant contained six histidine residues at the C-terminus (Section Materials and Methods, Table 1).

**Figure 3:**
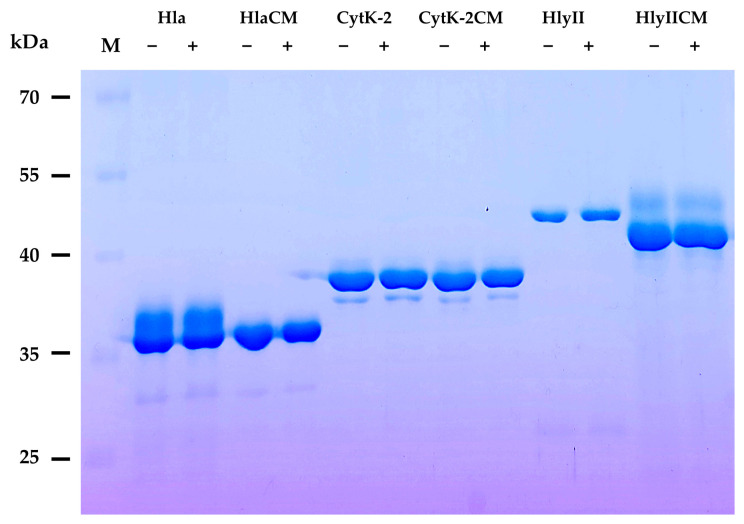
SDS-PAGE analysis of purified wild-type and mutant toxins under reducing and non-reducing conditions. For each protein, two samples are shown: “−” indicates treatment in the absence of 2-mercaptoethanol (non-reducing), and “+” is the treatment in the presence of 5% (*v*/*v*) 2-mercaptoethanol (reducing). Lane M contains molecular weight markers with sizes in kDa.

The TEV and thrombin cleavage sites were included to allow for optional removal of the affinity tags if required for future structural studies; however, tags were not removed in the present work. The resulting mutant proteins lacked a signal peptide and were expressed in the cytoplasm of *Escherichia coli* cells. The proteins were purified to near homogeneity by affinity chromatography in the presence of 8 M urea (Figure 3). Molecular weights were confirmed by SDS-PAGE and were consistent with those calculated.

In the absence of a reducing agent, purified mutant proteins migrated slightly faster than in the presence of a reducing agent, suggesting possible formation of disulfide bonds, which results in the protein becoming more compact and migrating faster during electrophoresis (Figure 3). The mobility of wild-type proteins did not change in the presence or absence of a reducing agent. These results are consistent with previous studies on Hla, where introduction of cysteine residues also leads to a mobility shift under non-reducing conditions, confirming the formation of intramolecular disulfide bonds [42].

**Figure 4:**
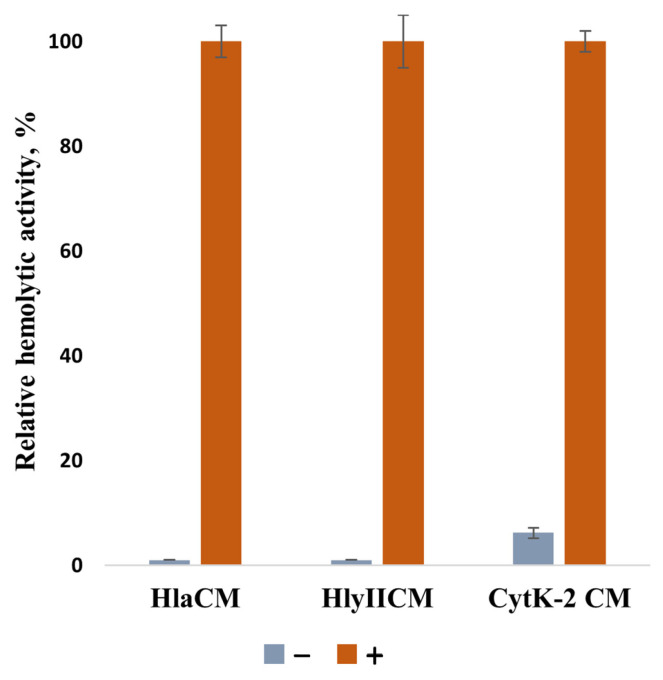
Relative hemolytic activity of HlaCM, CytK-2CM, and HlyIICM in the absence or presence of 5 mM DTT. For each mutant, activity in the presence of 5 mM DTT was set to 100% (absolute values: HlaCM—500 HU/mL; CytK-2CM—500 HU/mL; HlyIICM—1000 HU/mL). In the absence of DTT, the activity of HlaCM and HlyIICM did not exceed the background erythrocyte lysis levels. Wild-type toxins exhibited no change in activity upon DTT addition (Hla—300 HU/mL; CytK-2—1000 HU/mL; HlyII—2000 HU/mL). Data represent the mean of three independent experiments; error bars indicate standard deviation (S.D.).

Based on the structural prediction, we hypothesize that in the absence of a reducing agent, disulfide bonds form in mutant proteins. Their three-dimensional structures may then be unable to complete pore formation due to the change in the distance between Cβ atoms of the introduced cysteine residues when comparing the monomeric and oligomeric forms (Figure 1b). Consequently, we hypothesize that these mutants would exhibit no hemolytic activity in the absence of a reducing agent, because the monomer would contain an intramolecular disulfide bond that prevents pore formation. Hemolytic activity should be restored upon addition of a reducing agent. To test their hemolytic activity, mutant proteins were incubated with rabbit red blood cells in the presence or absence of a reducing agent, and the increase in absorbance at 490 nm due to hemoglobin release was monitored (Figure 4). In the absence of a reducing agent, the mutant proteins showed no hemolytic activity above the background levels. The absence of a hemolytic activity of cysteine mutants may indicate that most of these toxin molecules are capable of forming disulfide bonds during purification, thereby blocking pore formation.

Mutant forms of the toxins HlaCM, HlyIICM, and CytK-2CM demonstrated hemolytic activity in the presence of a reducing agent. CytK-2CM exhibited a residual activity in the absence of a reducing agent, suggesting that a minor portion of the mutant protein did not form a disulfide bond. Mutant forms of HlyIICM and HlaCM lack any hemolytic activity in the absence of a reducing agent, indicating that all cysteine residues are involved in S-S bond formation. Despite the fact that the proteins in the experiments had different concentrations and hemolytic activities, treatment with a reducing agent (DTT) restored the hemolytic activity to a level close to that of the toxins without mutations. These data indicate that the introduced cysteine residues do not disrupt the 3D structure of the studied toxins (Figure 4). Importantly, the presence of affinity tags, namely the N-terminal hexahistidine tag in HlyIICM and the C-terminal hexahistidine tags in HlaCM and CytK-2CM, did not interfere with the recovery of hemolytic activity upon reduction, confirming that the tags do not prevent proper folding and function. The cytolytic activity of toxins that contain no cysteine residues is not susceptible to the presence of SH reagents.

### 2.2. Survival of Experimental Animals After Intravenous Injections with Wild-Type and Mutant Forms of Toxins

The toxic activity of wild-type HlyII, CytK-2, and Hla and their mutant forms (HlyIICM, CytK-2CM, HlaCM) was assessed in BALB/c mice. In the control groups, animals were tested intravenously with a dose of the corresponding wild-type toxin equal to LD_50_: 35 HU HlyII (8 μg/mouse), 2.5 HU Hla (3 μg/mouse), 18 HU CytK-2 (1 μg/mouse). Mutant forms were also tested intravenously, at a dose corresponding to the LD_50_ of wild-type toxins. After 48 h of observation, all mice injected with mutant forms of toxins remained alive. In each control group, 50% of the animals that received the intravenous injection of toxins were alive. Thus, the results obtained in experimental animals are consistent with the level of hemolytic activity of the mutant forms of the toxins.

### 2.3. Immunization of Animals with Mutant Forms and Analysis of Immune Sera

Immunization of experimental mice with mutant forms of toxins tested their potential as vaccine prototypes. The titer of the immune serum was determined as the minimum dilution at which binding to the toxin was twice the background value. The results are presented in Table 2. Under identical experimental conditions, the lowest titers against HlyII were observed after immunization with HlyIICM. Immune sera following immunization with HlaCM and, to a greater extent, CytK-2CM demonstrated the highest levels of class G antibodies interacting with Hla and CytK-2, respectively. These results indicate the possibility of using mutant forms of bacterial pore-forming proteins to produce monoclonal antibodies to full-length toxins. In the case of immunization with HlyIICM, the relative content of class M antibodies in the immune sera was higher relative to the content of specific class G antibodies compared to immunization with HlaCM and CytK-2CM. Immunization with HlaCM and CytK-2CM leads to a greater degree of immunological memory formation.

### 2.4. Protective Ability of Immune Sera In Vitro and In Vivo

**Figure 5:**
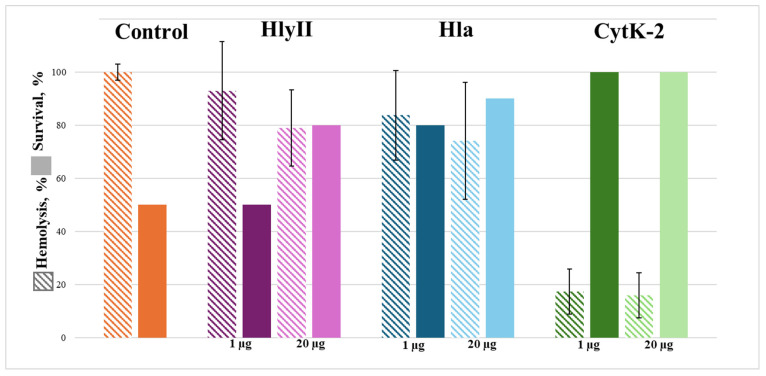
Inhibition of hemolytic activity of toxins in vitro, showing the percentage of immune sera after immunization with doses of 1 and 20 μg/mouse (inhibition data are presented as mean ± SD, *n* = 20); survival of immunized mice after administration of the LD_50_ toxin (data are presented as the percentage of surviving animals out of the total number of animals in the group, *n* = 10).

The effectiveness of hemolysis suppression by immune sera obtained by immunizing mice with mutant forms of the HlyII, Hla, and CytK-2 toxins was different. In the case of CytK-2CM immunization, all sera obtained against this toxin inhibited erythrocyte hemolysis regardless of the toxin dose injected. Hemolysis suppression by these sera was greater than 80%. Almost all sera obtained from animals immunized with HlaCM inhibited hemolysis. The level of hemolysis inhibition was nearly the same regardless of the toxin dose injected and was less than 25%. Only four out of twenty sera obtained from animals injected with a 20 μg dose inhibited hemolysis by more than 50% (Figure 5). During immunization with HlyIICM, only the sera with specific IgG titers greater than 1/500,000 showed the ability to inhibit hemolysis (Table 2). After immunization with a dose of 20 μg/mouse, 3 out of 20 such sera were detected. Thus, increasing the dose of HlyIICM and HlaCM antigens during immunization to 20 μg per animal increased the protective capacity of the immune sera (Figure 5).

The protective effect of in vivo immunization of experimental animals with mutant forms of the studied toxins was tested by intravenous injection of the toxins into immunized animals at a dose of LD_50_. Mice immunized with HlyIICM were injected with HlyII; those immunized with HlaCM, with Hla; and those immunized with CytK-2CM, with CytK-2. The results are shown in the diagram in Figure 5. The lowest survival values were demonstrated by sera from mice immunized with HlyIICM at a dose of 1 μg per mouse. Increasing the HlyIICM dose to 20 μg/mouse increased the mouse survival. When HlaCM was used as the antigen, the survival was higher but did not reach 100%. All mice immunized with CytK-2CM remained alive after injection with CytK-2 at LD_50_, regardless of the toxin dose used at immunization. Survival data correlate with data on specific antibody levels and the ability of immune sera to inhibit hemolysis in vitro (Table 2 and Figure 5).

Among the mutant forms studied, only CytK-2CM exhibited the properties of the vaccine prototype: immune sera completely protected experimental animals from the effects of CytK-2 at intravenous injection.

## 3. Discussion

Alterations in the toxicity of pore-forming toxins can be achieved by limiting the conversion of the water-soluble monomeric form to a membrane-bound oligomer. A possible way to do this is to influence the formation of the toxic oligomeric form. From the moment of synthesis on the ribosome to the lysis of target cells, the protein undergoes several conformational rearrangements [10,11]. Initially, in the bacterial cell, the protein exists in a soluble monomeric form. Subsequent conformational rearrangements occur upon its secretion through the bacterial membrane and upon binding to the membrane of a target cell. Previously, studies have been conducted on neutralizing antibodies obtained against individual fragments of pore-forming toxins, as the use of full-length toxins to produce monoclonal antibodies is significantly limited due to their toxicity [26,28,29,30]. The use of mutant forms of pore-forming toxins for these purposes is also limited because the loss of cytolytic properties is often associated with irreversible structural changes [31,32]. In this study, we propose a variant of toxin structural mutations that can restore the activity under certain conditions. Thus, the injection of non-toxic cysteine mutants into animals ensures the formation of antibodies against a wide range of epitopes.

HlaCM, HlyIICM, and CytK-2CM toxins are stable in the monomeric state. A significant portion of these proteins was insoluble and was found in cell debris, while a small amount of the toxins was present in the cytosol as a water-soluble fraction. Therefore, the purification of the mutant toxins from the debris was carried out in the presence of 8 M urea. Purified preparations were used for further experiments with at least a 400-fold dilution, which ensured the recovery of the toxin structure and activity. At this dilution, both wild-type and mutant toxin preparations were used to carry out a comparative analysis of the efficiency of hemolysis in the presence and absence of reducing agents. In the presence of SH reagents, the mutant proteins exhibited hemolytic activity. Therefore, the introduced cysteines did not change the toxins’ 3D structures, confirming our hypothesis about the positions of cysteine residues, which allows the formation of a disulfide bond in the monomers and prohibits pore formation. Direct electrophoretic confirmation of the blockade of mature functional toxin formation is complicated by the short lifetime of intermediate oligomeric structures during pore formation. These intermediates exist either as small oligomers associated with the membrane or as structures partially embedded in the membrane [43,44]. At the first stage after the introduction of monomers and their diffusion along the lipid layer of the phospholipid membrane [2], monomers undergo numerous collisions, resulting in the formation of a transient dimer. The lifetime of the dimers is about 50 ms, after which they dissociate into monomers; reorganization of monomers with the transient complex leads to reversible stepwise growth. Although heptamer formation results in the creation of a stable complex that does not dissociate, the number of heptamers formed is tens of times less than the number of the original monomers since almost all intermediate complexes rapidly dissociate [12]. To visualize the transition of monomers into mature pores, it is necessary to ensure the fixation of intermediate states. At the same time, the presence of functional pores is necessary to obtain neutralizing antibodies. The possibility of transition of mutant forms of toxin monomers into functional pores is presumably determined by the presence of SH reagents. The mutant forms obtained in this work exhibited no hemolytic activity in vitro in the absence of SH reagents and were used for experiments with animals. This paper shows that mutant forms of bacterial β-PFTs with introduced cysteine residues are non-toxic and can be used to immunize mice as a prospect for obtaining monoclonal antibodies against the full-length monomeric form of β-barrel PFTs (e.g., Hla, CytK2, HlyII) and to develop vaccine prototypes. Immune sera after immunization with HlaCM and CytK2CM revealed high levels of IgG antibodies, compared to IgM, interacting with wild-type toxins, indicating the formation of immunological memory. The obtained immune sera were capable of suppressing the hemolytic activity of the wild-type toxins in vitro and in vivo.

## 4. Materials and Methods

### 4.1. Construction of Mutant Genes

Mutant forms of *hlyII* Q147C/V166C (*hlyIICM*), *hla* Q150C/V167C (*hlaCM*), and *cytK-2* Q156C/V175C (*cytK-2CM*) lacking the signal peptide were generated by the overlap extension PCR. Previously obtained plasmids carrying the wild-type genes [28,45] were used as templates. The PCR reactions were performed using Q5 High-Fidelity DNA Polymerase (New England Biolabs, Ipswich, MA, USA) according to the manufacturer’s protocol. The primers used for mutagenesis are listed in Table 3. The following cycling conditions were applied: initial denaturation at 98 °C for 30 s; 30 cycles of denaturation at 98 °C for 10 s, annealing at 50 °C for 30 s, and extension at 72 °C for 40 s; the final extension at 72 °C for 3 min. In the first step, two fragments were amplified, each containing one of the target mutations. In the second step, the fragments were combined to produce the full-length mutated sequence. The final PCR products were cloned into pET19mod (HlyII CM), pET28b (HlaCM), and pET29b (CytK-2 CM) using NdeI and XhoI restriction sites (Thermo Fisher Scientific, Waltham, MA, USA). The pET vectors were chosen because they enable high-level expression under the control of the T7 promoter in *E. coli* BL21(DE3). All constructs were verified by sequencing (Evrogen, Moscow, Russia). Recombinant plasmids were transformed into *E. coli* BL21(DE3) for protein expression. The resulting proteins, designated HlaCM, HlyIICM, and CytK-2CM, contained affinity tags as indicated in Table 1.

### 4.2. Protein Expression and Purification

*E. coli* BL21(DE3) cells harboring the recombinant plasmids were grown in 150 mL of Luria–Bertani (LB) medium containing 100 μg/mL ampicillin (for HlyIICM constructs) or 20 μg/mL kanamycin (for HlaCM and CytK-2CM constructs) at 37 °C with shaking until the optical density at 600 nm (OD_600_) reached 0.6–0.8. Protein expression was induced by adding isopropyl-β-D-1-thiogalactopyranoside (IPTG) to a final concentration of 0.1 mM, and cultivation was continued for 16 h at 20 °C.

The biomass was harvested by centrifugation at 4500× *g* for 15 min at 4 °C (Eppendorf 5810R centrifuge, Eppendorf, Hamburg, Germany). The cell pellet was resuspended in 12 mL of buffer B (100 mM sodium phosphate buffer pH 8.0, 10 mM Tris-HCl pH 8.0, 8 M urea) supplemented with 1 mM phenylmethylsulfonyl fluoride (PMSF). The cell suspension was subjected to sonication on ice using a QSonica Q700 ultrasonic homogenizer for seven 20 s cycles at 35% amplitude, with 2 min intervals between the cycles. The lysate was clarified by centrifugation at 17,000× *g* for 30 min at 4 °C.

The clarified lysate was applied to a 1 mL Ni-NTA agarose column (Qiagen, Hilden, Germany) pre-equilibrated with buffer B. The column was sequentially washed with 15 column volumes of buffer B and buffer C (100 mM sodium phosphate, 10 mM Tris-HCl, 8 M urea, 1 mM PMSF, adjusted to pH 6.3 with HCl). Bound proteins were eluted stepwise with buffer D (same composition as buffer C, adjusted to pH 5.9) and buffer E (adjusted to pH 4.5). All steps were performed at room temperature according to the manufacturer’s instructions for purification under denaturing conditions. Peak fractions containing the target proteins (typically eluted with buffer D) were collected and used for further analysis.

Protein purity and molecular weight were assessed by SDS-PAGE. To evaluate disulfide bond formation, samples were prepared in the presence or absence of 5% (*v*/*v*) 2-mercaptoethanol prior to electrophoresis. Gels were stained with Coomassie Brilliant Blue R-250.

### 4.3. Hemolysis Assay

The hemolysis assay was performed according to reference [42] with modifications as follows. Investigated proteins were adjusted with 1-M Tris-HCl buffer, from pH 8.0 up to pH 7.5. Probes were pretreated in the presence or absence of 10 mM dithiothreitol (DTT) in hemolysis buffer (10 mM Tris-HCl pH 7.4 in buffer saline) at room temperature for 10 min. Hemolysis reactions were then carried out according to reference [46]. One hemolytic unit was defined as the concentration of hemolysin required to achieve 50% lysis of erythrocytes.

### 4.4. Analysis of Experimental Animals’ Survival After Intravenous Injections with Wild-Type and Mutant Forms of Toxins

BALB/c mice aged 6–8 weeks, weighing about 20 g each, were used. The animals were obtained from the Laboratory Animal Nursery of the BIBCh RAS, Pushchino. Animal studies were approved by the Commission for the Control of the Maintenance and Use of Laboratory Animals of the BIBCh RAS (Protocol No. 998/24 dated 21 September 2024) and were conducted using all institutional rules for the care and use of animals. The functional activity of the toxin preparations was evaluated in HU. The LD_50_ for the studied toxins was determined by the Reed and Muench method [47].

### 4.5. Immunization with HlyIICM, HlaCM, and CytK-2CM

BALB/c mice aged 6–8 weeks, weighing about 20 g each, obtained from the Laboratory Animal Nursery of the BIBCh RAS, Pushchino, were used. Animal studies were approved by the Commission for the Control of the Maintenance and Use of Laboratory Animals of the BIBCh RAS (Protocol No. 998/24 dated 21 September 2024). Immunization with recombinant mutant forms of the toxins was performed intraperitoneally at two-week intervals: the first immunization in complete Freund’s adjuvant, the second and third immunizations in incomplete Freund’s adjuvant. Each of the preparations (HlyIICM, HlaCM, and CytK-2CM) was injected at doses of 1 and 20 μg per animal. Each experimental group consisted of 20 animals. Blood samples for analysis were collected from the tail vein on the 7th day after the third immunization. Normal (non-immune) mouse serum at the same dilution was used as a negative control.

### 4.6. Inhibition of the Hemolytic Activity of HlyII, Hla, and CytK-2 by Immune Sera In Vitro

The reaction was carried out in PBS, in a volume of 100 μL. Each reaction mixture was pre-incubated for 1 h with the appropriate hemolysin at a concentration required to achieve 50% erythrocyte lysis and with the analyzed immune serum at a 1/50 dilution. Normal mouse serum at the same dilution was added to control samples. Rabbit erythrocytes were then added to a concentration of 1%. The suspension was incubated for 1 h at RT.

### 4.7. Survival of Immunized Animals After Intravenous Injection of Functionally Active Toxins

Toxins at a dose of LD_50_ were administered via the tail vein of experimental animals in 100 μL of PBS on the 9th day after the third immunization. Three groups, each consisting of 10 non-immunized animals, were used as controls. Animals in the first control group were injected with HlyII; in the second, with Hla; and in the third, with CytK-2, each at a dose of LD_50_. The animals were observed for 48 h, noting the incidence of death. Mice had free access to food and water throughout the experiment.

### 4.8. Enzyme-Linked Immunosorbent Assay

The relative content of specific antibodies, i.e., those recognizing HlyII, Hla, and CytK-2, in the sera of immunized animals was determined using indirect solid-phase ELISA based on the interaction of immune sera with the corresponding test toxins immobilized at a concentration of 1 μg/mL in 0.05 M carbonate buffer, pH 9.6. Immune sera were titrated in 2-fold increments, starting with a dilution of 1/2000. To determine the relative content of IgG interacting with the corresponding toxin, goat anti-mouse IgG [H+L] conjugated with horseradish peroxidase (Thermo Scientific, Waltham, MA, USA) was used in dilution according to the manufacturer’s instructions (1 h at 37 °C). The content of specific IgM was determined by sequential addition of goat anti-mouse IgM antibodies (Abcam, Waltham, MA, USA) and a conjugate of rabbit anti-goat immunoglobulins with horseradish peroxidase (Imtek, Moscow, Russia). A PBST solution (PBS containing 0.1% Tween 20) was used for: blocking free binding sites on the plate surface; diluting sera and conjugates; washing plates between steps. The reaction was visualized by adding *ortho*-phenylenediamine peroxidase substrate.

## 5. Conclusions

This study showed that introduction of cysteine residues into the homologous region of bacterial β-PFTs significantly reduced both their hemolytic activity against rabbit erythrocytes and their toxigenic activity upon injection into experimental animals. The restoration of hemolytic activity in the mutant toxins treated with a reducing agent was consistent with the hypothesis that the disulfide bond is responsible for the loss of function. Immunization with the mutant toxins increased the survival of animals subsequently challenged with wild-type toxins. In particular, CytK-2CM can be used as a candidate antigen for the development of a vaccine prototype.

## Figures and Tables

**Table 1:** Characteristics of wild-type and mutant toxin constructs.

Protein	Description/Affinity Tags	Calculated MW (kDa)
HlyII	C-terminal thrombin site and 6xHis tag	46.3
HlyIICM	N-terminal 6xHis and TEV protease cleavage site	44.9
Hla	C-terminal 6xHis tag	34.3
HlaCM	C-terminal 6xHis tag	34.2
CytK-2	C-terminal thrombin site and 6xHis tag	37.4
CytK-2CM	C-terminal thrombin site and 6xHis tag	37.4

**Table 2:** Relative content of immunoglobulins in the sera of animals immunized with mutant toxins interacting with the corresponding toxin (titer).

Dose of Mutant Toxin/Mouse	Range of Titer Values IgG (*n* * 1000)	Titers IgM (*n* * 1000) for Interaction with			% of Sera from the Total Amount in the Group (*n* = 20), Interacting with		
		HlyII	Hla	CytK-2	HlyII	Hla	CytK-2
1 µg	1/16–1/64	1/8–1/64	n/d	n/d	55	0	0
	1/128–1/256	1/16–1/64	1/12–1/24	n/d	30	40	0
	1/256–1/512	1/32	1/12–1/32	1/32–1/64	15	45	55
	>1000	n/d	1/32–1/48	1/64	0	15	45
20 µg	1/32–1/64	1/8–1/16	n/d	n/d	25	0	0
	1/128–1/256	1/16–1/32	1/48	n/d	45	5	0
	1/256–1/512	1/32–1/64	1/32–1/96	1/32–1/64	15	20	25
	>1000	1/64–1/128	1/48–1/384	1/64	15	75	75

* n/d—not detectable.

**Table 3:** Primers.

Gene Name	Primer Name	Primer Sequence
*hlyIICM*	F1_hlyII	TAATTTCATATGGATTCTAAAGGAACTGTAGA
	R1_hlyII	CTGTGTGGGTCTCTAATACCGTTTTATAATCTACGCAATCATAAGATAC
	F2_hlyII	CGGTATTAGAGACCCACACAGATAAAAAATTAAATTGGAAATGCGGATTCCAATC
	R2_hlyII	TAATACTCGAGTCAGATCTGTTTAATCTCGATA
*hlaCM*	F1_hla	TAATACGACTCACTATAGG
	R1_hla	CAGTTGGGCTCTCTAAAATTGTTTTGAAATCAGGGCAAACATATTTCAG
	F2_hla	CAATTTTAGAGAGCCCAACTGATAAAAAAGTAGGCTGGAAATGCATATTTAACAATATG
	R2_hla	AAGGGGTTATGCTAGTTA
*cytK-2CM*	F1_cytK-2	TTATAGGATCCCATATGCAAACGACGTCACAAG
	R1_cytK-2	CGTTTGGTCAATTAAATTTGTTTTATAACTAGTGCATTTATAGCTTACAGAG
	F2_cytK-2	CAAATTTAATTGACCAAACGAACAAAAACGTAAAATGGAACTGCTTCTTTAACGGATATAAC
	R2_cytK-2	TTACTCGAGGGTACCTTTTTTCTCTACCAATTTCTTATTC

## Data Availability

The original contributions presented in this study are included in the article. Further inquiries can be directed to the corresponding authors.
